# Multilocus microsatellite typing (MLMT) reveals host-related population structure in *Leishmania infantum* from northeastern Italy

**DOI:** 10.1371/journal.pntd.0006595

**Published:** 2018-07-05

**Authors:** Gianluca Rugna, Elena Carra, Federica Bergamini, Mattia Calzolari, Daniela Salvatore, Francesco Corpus, William Gennari, Raffaella Baldelli, Massimo Fabbi, Silvano Natalini, Fabrizio Vitale, Stefania Varani, Giuseppe Merialdi

**Affiliations:** 1 Istituto Zooprofilattico Sperimentale della Lombardia e dell’Emilia-Romagna, Brescia, Italy; 2 Department of Veterinary Medical Sciences, University of Bologna, Ozzano Emilia, Italy; 3 Dipartimento Integrati Interaziendali Medicina di Laboratorio e Anatomia Patologica, Azienda Ospedaliero-Universitaria, Policlinico di Modena, Italy; 4 Department of Animal Health, Azienda Unità Sanitaria Locale, Bologna, Italy; 5 National Reference Center for Leishmaniasis (C.Re.Na.L.), Istituto Zooprofilattico Sperimentale della Sicilia, Palermo, Italy; 6 Unit of Clinical Microbiology, Regional Reference Centre for Microbiological Emergencies (CRREM), St. Orsola-Malpighi University Hospital, Bologna, Italy; 7 Department of Experimental, Diagnostic and Specialty Medicine, University of Bologna, Italy; Institut de Recherche pour le Développement, FRANCE

## Abstract

**Background:**

Visceral leishmaniasis (VL) caused by *Leishmania infantum* is an ongoing health problem in southern Europe, where dogs are considered the main reservoirs of the disease. Current data point to a northward spread of VL and canine leishmaniasis (CanL) in Italy, with new foci in northern regions previously regarded as non-endemic.

**Methodology/Principal findings:**

Multilocus microsatellite typing (MLMT) was performed to investigate genetic diversity and population structure of *L*. *infantum* on 55 samples from infected humans, dogs and sand flies of the E-R region between 2013 and 2017. E-R samples were compared with 10 *L*. *infantum* samples from VL cases in other Italian regions (extra E-R) and with 52 strains within the *L*. *donovani* complex. Data displayed significant microsatellite polymorphisms with low allelic heterozygosity. Forty-one unique and eight repeated MLMT profiles were recognized among the *L*. *infantum* samples from E-R, and ten unique MLMT profiles were assigned to the extra E-R samples. Bayesian analysis assigned E-R samples to two distinct populations, with further sub-structuring within each of them; all CanL samples belonged to one population, genetically related to Mediterranean MON-1 strains, while all but one VL cases as well as the isolate from the sand fly *Phlebotomus perfiliewi* fell under the second population. Conversely, VL samples from other Italian regions proved to be genetically similar to strains circulating in dogs.

**Conclusions/Significance:**

A peculiar epidemiological situation was observed in northeastern Italy, with the co-circulation of two distinct populations of *L*. *infantum*; one population mainly detected in dogs and the other population detected in humans and in a sand fly. While the classical cycle of CanL in Italy fits well into the data obtained for the first population, the population found in infected humans exhibits a different cycle, probably not involving a canine reservoir. This study can contribute to a better understanding of the population structure of *L*. *infantum* circulating in northeastern Italy, thus providing useful epidemiologic information for public health authorities.

## Author summary

Visceral leishmaniasis is a sand fly-borne disease caused by protozoan parasites of the genus *Leishmania*. *Leishmania infantum* is the only parasitic species circulating in Italy and dogs are considered the main reservoirs of the disease. In this study, 55 *L*. *infantum* strains obtained from humans, dogs and sand flies from the Emiliana-Romagna (E-R) region, northeastern Italy, were assessed using multilocus microsatellite typing, a tool applied for population genetic studies. Results were compared with those obtained from 10 samples of visceral leishmaniasis cases occurring in other Italian regions and with 52 strains of the *L*. *donovani* complex from other foci of leishmaniasis. Our genetic analysis revealed that canine and human *L*. *infantum* strains from the E-R region were separated in two distinct populations; all samples obtained from dogs belonged to one population, while all but one human samples as well as a sand fly sample fell under another population. Samples from patients with visceral leishmaniasis from other Italian regions proved to be genetically similar to strains circulating in dogs. Our findings raise questions on the role of dogs as main reservoirs for human visceral leishmaniasis in the investigated area of northeastern Italy.

## Introduction

Visceral leishmaniasis (VL) is a vector-borne systemic disease caused by protozoan parasites of the *Leishmania donovani (L*. *donovani)* complex. VL is endemic in the Mediterranean Europe, where the disease is caused by *Leishmania infantum (L*. *infantum)* and the infection occurs through the bite of infected female phlebotomine sand fly species of the genus *Phlebotomus* [1]. In this area, dogs are considered the principal domestic reservoirs of VL [2].

In the last decades, the incidence of VL and canine leishmaniasis (CanL) increased in Italy, with new foci within traditional endemic areas and with a spread of leishmaniasis to northern regions previously regarded as non-endemic [3–5]. As an example, a remarkable increase of VL cases has been recently reported in the Emilia-Romagna (E-R) region, which is located in northeastern Italy [6,7]. Following this increase, the surveillance tasks that were previously implemented for CanL in public kennels of the E-R region [8] have been extended to humans and vectors and included a molecular surveillance of circulating *Leishmania* strains. The evaluation of genetic markers, such as the repetitive nuclear region on chromosome 31, the *cpb*E/F-gene and the *k26*-gene led to the observation that *L*. *infantum* strains circulating in the E-R region exhibit genetic polymorphism [9]. Molecular markers capable of resolving genetic differences to strain level are useful to understand parasite’s biology and dynamics of disease [10] and their employment can generate molecular epidemiology data, which may provide crucial information for public health authorities. Different genotyping methods have been developed for population genetic studies of the *L*. *donovani* complex [11]. Among others, multilocus microsatellite typing (MLMT), based on length variations of 14–15 co-dominant microsatellite markers, has proven to be a powerful tool to discriminate strains, thus giving useful insights into the epidemiology of leishmaniasis [12–14].

In this study, a selected panel of 15 previously described microsatellite markers [12,13,15] was employed to investigate the genetic structure and gene diversity among strains of *L*. *infantum* obtained from cases of VL, CanL, and from infected phlebotomine sand flies of the E-R region. The obtained profiles were compared with those of other *L*. *infantum* strains from Italy and with available MLMT profiles [16–19], mainly from the Mediterranean Basin, to determine their genetic inter-relationship.

## Methods

### Origin of isolates and biological samples

**Fig 1:**
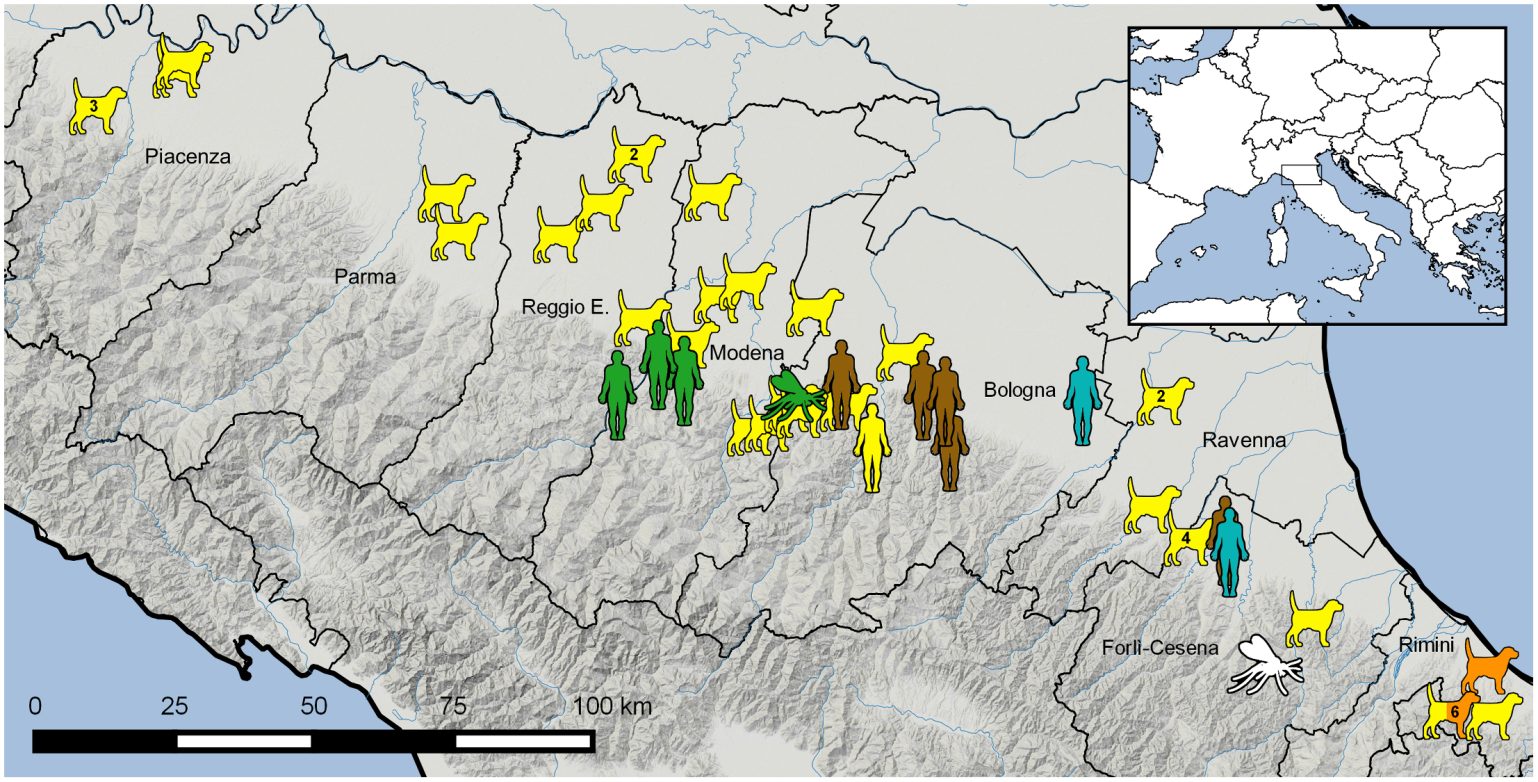
Geographical distribution of human, canine and sand fly *Leishmania*-positive samples, 2013–2017, Emilia-Romagna region (northeastern Italy). Colors depict the subpopulations inferred by STRUCTURE analysis: yellow for subPopA1; orange for subPopA2; green for subPopB1; brown for subPopB2; light blue for subPopB3; white for samples not submitted to cluster analysis. More than one sample per area are shown by numbers inside the icon. Map generated with Quantum-GIS (https://www.qgis.org/it/site/).

A panel of 65 *Leishmania* samples (53 isolates and 12 DNA from biological samples) were included in the study. Samples were divided into two subsets (Table 1). The first subset, called E-R, included a total of 55 samples; 40 samples (38 isolates and 2 lymph node aspirates) obtained from CanL cases, 11 samples (4 isolates, 5 peripheral blood and 2 bone marrow aspirates) from VL cases, and 4 from sand flies. Samples were obtained from different provinces of the E-R region (Fig 1) during 2013–2017, with the exception of a cryopreserved canine isolate collected in 1998. Sand fly samples were composed of an isolate from one female of *Phlebotomus* (*Ph*.) *perfiliewi* and 3 sand fly pools collected in two different sites (Fig 1) by attractive traps baited with CO_2_. The second subset, called extra E-R, comprised 10 isolates obtained from VL patients that were admitted to the San Matteo Hospital between 2007 and 2011 (Pavia, Italy), and that were infected in different Italian regions (Calabria, Campania, Liguria, Lombardy, Tuscany and Sicily). The designation and characteristics of all samples included in this study are listed in S1 Table.

**Table 1:** Characteristics of *Leishmania*-positive samples and WHO reference strains included in the study.

Subset’s denomination (sample size)	Host	Number of samples (*i*, *d*)	Geographical origin (region)	N° of MLMT profiles
**E-R (55)**	Dogs	38 (i)	Italy (Emilia-Romagna)	29
		2 (d)		
	Humans	4 (i)	Italy (Emilia-Romagna)	9
		7 (d)		
	Sand flies	1 (i)	Italy (Emilia-Romagna)	4
		3 (d)		
**Extra E-R (10)**	Humans	10 (i)	Italy (Calabria, Campania, Liguria, Lombardy, Tuscany, Sicily)	10
**WHO reference strain (zymodeme)**				
MHOM/TN/80/IPT1 (MON1)			Tunisia	
MHOM/IT/86/ISS218 (MON-72)			Italy	
MHOM/DZ/82/LIPA59 (MON-24)			Algeria	

E-R, Emilia-Romagna; i: isolates; d: DNA from biological samples

The MLMT profiles of the two subsets were compared with each other and with previously published profiles of 49 strains of the *L*. *donovani* complex [16–19] (S2 Table). Three WHO strains, namely *L*. *infantum* MHOM/TN/1980/IPT1 (MON-1), *L*. *infantum* MHOM/DZ/82/LIPA59 (MON-24) and *L*. *infantum* MHOM/IT/86/ISS218 (MON-72)—chosen as representative of zymodemes causing VL in Italy [5,20]—were typed in this study and also used for comparison (Table 1).

### DNA extraction

DNA from all isolates was obtained as previously described [9]. DNA from canine bone marrow/lymph node aspirates and from sand fly samples was extracted using Qiagen DNeasy Blood & Tissue Kit (Qiagen, Hilden, Germany) according to the manufacturer’s instructions, and the DNA was eluted in a final volume of 100 μl of elution buffer.

For VL cases, DNA was extracted from 100 μL whole blood (peripheral blood) or 100 μL bone marrow aspirate using NucliSENSeasyMAG (bioMerieux, Marcy l’Etoile, France).

### ITS-1 species typing

Twenty-five of the 65 samples included in the study were submitted to species identification by amplification and sequencing of the Internal Transcribed Spacer-1 (ITS-1) region according to El Tai et al. [21], and following the protocols described by Rugna et al. [9]. The results were compared to those of the other 40 samples that had been previously identified as *L*. *infantum* (GenBank accession numbers: KX096612-KX096651) [9].

### Multilocus microsatellite typing (MLMT)

A set of 15 dinucleotide microsatellite markers (Li41-56, Li46-67, Li21-34, Li22-35, Li23-41, Lm2TG, Lm4TA, Li71-5/2, LIST7039, Li71-33, Li71-7, CS20, Li45-24, TubCA and LIST7031), previously found highly discriminative within the *L*. *donovani* complex [12,13,15], were investigated in the present study. The amplification reaction of the microsatellite loci was performed as described elsewhere [22], using fluorescent-labeled forward primers (WellRed dyes, Sigma-Aldrich, Saint Louis, USA), and employing the described annealing temperature (Ta) except for loci LIST7039 (Ta = 54°C), CS20 (Ta = 56°C) and LIST7031 (Ta = 54°C). The amplicons were subjected to automated fragment analysis on the capillary CEQ 8000 sequencer (Beckman Coulter Inc., Indianapolis, USA) and the fragment sizes were determined using the “Fragment” package of the CEQ 8000 version 8.0 software.

The microsatellite repeat numbers were calculated for all loci by comparing the sizes of the respective fragments to those of the strain MHOM/TN/1980/IPT1, which was included in each run as a standard for fragment size, and for which the repeat numbers had been determined by direct sequencing (S1 Protocol) (AN: MG463110-MG463119).

The repeat numbers for all markers were then assembled into a multilocus microsatellite profile (MP) for each sample under study.

### Population structure and phylogenetic analysis

The multilocus genotype data were analyzed by using the Bayesian model-based clustering algorithm implemented in STRUCTURE v.2.3.4. software [23], which determines genetically distinct populations on the basis of allele frequencies and estimates the individual’s membership co-efficient (Q-value) in each probabilistic population. The Markov chain Monte Carlo iterations were set at 200,000 and the length of burn-in period at 20,000. For each value of *K* (estimated number of populations) between 1 and 10, a series of 10 iterations was performed to estimate values of Δ*K* [24], which were implemented on STRUCTURE HARVESTER v0.6.1 [25]. The CLUMPP 1.1.2 software [26] was used to deal with the “label switching” performing alignments of the Q-values for the chosen number of populations. The barplots of the CLUMPP outfiles were visualized using an online tool, STRUCTURE PLOT [27]. This clustering approach was used for the population genetic analyses by assigning individuals to the single cluster, in which they exhibited the highest Q-value. The distribution of genetic variation was evaluated using a Factorial Correspondence Analysis (FCA) implemented in GENETIX 4.05 [28]. Genetic relationships among samples were evaluated by phylogenetic trees that were constructed based on the Sainudiin model [29] using the application BEASTvntr package implemented in the BEAST2 2.4.3 software [30]. The diploid data were entered as two distinct partitions, with a linked tree and strict clock, and a chain length of ten million steps was set. Trees were visualized with FIGTREE 1.4 (http://tree.bio.ed.ac.uk/software/figtree/). Descriptive statistics for microsatellite markers and genetic populations were calculated by the GDA software [31]; this included allelic diversity (number of allelic variants per marker (A) and mean number of alleles (MNA) per population), proportion of polymorphic loci (P), expected (He) and observed (Ho) heterozygosity and inbreeding coefficient (F_is_). Genetic differentiation and gene flow were assessed by F-statistics calculating the F_st_ values [32] using MSA software [33]. F_st_ values higher than 0.25 with significant p-values (<0.05) indicated strong genetic differentiation [34]. Migration rates (Nm) were calculated as Nm = 0.25(1-F_st_)/F_st_ [14]. A MP frequency value—the ratio number of MLMT profiles/number of strains/number of markers—was determined for comparison of our results with other studies using the same microsatellites [18].

### Ethical clearance

Samples from VL cases in the E-R region were collected for diagnostic purposes; storing at -80 °C was carried out at the Regional Reference Centre for Microbiological Emergencies (CRREM), St. Orsola-Malpighi University Hospital (Bologna, Italy), while MLMT evaluation was performed at the Istituto Zooprofilattico Sperimentale della Lombardia e dell’Emilia-Romagna, Modena unit (Italy). Storing and molecular evaluation of retrospectively collected samples received the approvals from both the Ethics Committee of the St. Orsola-Malpighi University Hospital, Bologna, Italy (protocol No. 3729/2017) and the Modena Ethics Committee, Modena, Italy (protocol No. 239/13). Samples were coded and anonymized.

Extra E-R *Leishmania* isolates derived from a pre-existing cryobank of the Fondazione IRCCS Policlinico S. Matteo, Pavia, Italy. *Leishmania* reference strains were obtained from the collection hosted by the National Reference Center for Leishmaniasis, Palermo, Italy.

All animal samples were obtained for diagnostic purpose with no unnecessary invasive procedures, as part of the Emilia-Romagna surveillance program on leishmaniasis, including parasitological confirmation of CanL in clinically suspected dogs. Evaluation of these samples did not require ethical approval according to the European Directive 2010/63/EU. Oral informed consent was obtained from the owners of dogs at the time of clinical examination.

### Accession numbers

MG463110-MG463119

## Results

The ITS-1 sequences obtained in this study for 25 samples showed 100% identity with those of 40 samples previously identified as *L*. *infantum* [9]. In total, 65 *L*. *infantum* samples were submitted to microsatellite typing.

### Microsatellite variation in *L*. *infantum* strains from the E-R region

Fifteen microsatellite polymorphic loci were used to assess the genetic variation of the 55 *L*. *infantum* samples obtained from VL (n = 11), CanL (n = 40) and sand flies (n = 4) from the E-R region. Amplification reactions were successfully performed for 54 samples, while the sample SF2 resulted negative for the locus Li23-41. All genotyped samples showed one or two peaks in the electropherogram, suggesting that there was no occurrence of aneuploidy (S1 Table). All markers but one (Li71-33) were polymorphic, showing between 2 and 10 different alleles per marker. The most variable markers were Lm2TG (10 alleles) and Lm4TA (8 alleles), while Li41-56, Li46-67 and Li71-5/2 were the least polymorphic, presenting only 2 alleles for each marker. The mean number of allelic variants per locus (A) was 4.33 (Table 2). Heterozygosity was seen in 11 loci. The observed heterozygosity (Ho) was between 0.000 and 0.200 and the expected heterozygosity (He), representing the probability that an individual will be heterozygous over the loci tested, ranged from 0.000 to 0.825 and it was in all cases higher than Ho, with the exception of locus Li71-33 (Ho = 0.000). Inbreeding coefficient per locus (F_is_) was positive for 14 markers and ranged from 0.727 to 1.000 (mean 0.869); for just one marker (Li71-33), the F_is_ value was zero. Taken together, these findings indicate a high degree of homozygosis in the investigated samples (Table 2).

**Table 2:** Genetic characteristics and variation of the 15 microsatellite loci detected in the population of *Leishmania infantum* from the Emilia-Romagna region (northeastern Italy, N = 55).

Locus	N	Repeat array	Fragment size array (bp)	A	He	Ho	F_is_
Li41-56	55	CA (9–10)	90–92	2	0.224	0.000	1.000
Li46-67	55	CA (7–10)	74–80	2	0.383	0.000	1.000
Li21-34	55	CA (8–16)	81–97	4	0.441	0.018	0.959
Li22-35	55	CA (9–19)	86–106	5	0.659	0.091	0.863
Li23-41	54	GT (12–17)	77–87	4	0.471	0.130	0.727
Lm2TG	55	TG (9–27)	110–146	10	0.825	0.200	0.759
Lm4TA	55	TA (9–20)	73–95	8	0.803	0.145	0.820
Li71-5/2	55	CA (8–9)	108–110	2	0.036	0.000	1.000
LIST7039	55	CA (13–17)	203–211	4	0.595	0.036	0.939
Li71-33	55	TG (11)	105	1	0.000	0.000	0.000
Li71-7	55	CA (7–13)	88–100	6	0.539	0.127	0.766
CS20	55	TG (15–21)	75–87	5	0.634	0.036	0.943
Li45-24	55	CA (7–16)	89–107	6	0.706	0.109	0.847
TubCA	55	CA (9–13)	80–88	3	0.414	0.018	0.956
LIST7031	55	CA (8–11)	165–171	3	0.455	0.036	0.921
**Overall**	**55**			**4.33**	**0.479**	**0.063**	**0.869**

Locus, analyzed microsatellite marker; N, sample size; Repeat array per locus, in brackets the overall identified dinucleotide repeat numbers; Fragment size array per locus, the overall fragment sizes are indicated; A, number of alleles; He, expected heterozygosity; Ho, observed heterozygosity; F_is_, inbreeding coefficient.

In total, 41 different multilocus microsatellite profiles (MP) were assigned to 55 *L*. *infantum* samples (Table 1 and S1 Table), giving a MP frequency (profiles per sample per locus) of 0.050. Homozygous allele combination predominated in the examined samples; 18 out of 41 were di-allelic in at least one locus and were deemed as heterozygous. Thirty-two out of 41 MPs were unique to individual samples and 8 (MP6, MP7, MP8, MP19, MP21, MP22, MP23, MP38) were shared by more than one sample (S1 Table). Among 8 repeated MPs, 6 were shared by small groups of isolates (from 2 to 5) within the same focus of leishmaniasis. In detail, MP6 and MP7 were isolated in the Forlì-Cesena province, MP19 in the Rimini province, MP21 in the Piacenza province, MP22 in the Modena province, and MP23 in the Bologna province. The remaining 2 repeated MPs were shared by isolates from distinct areas; 3 strains of MP8 were isolated from dogs living in distant provinces of E-R (i.e. Ravenna and Reggio Emilia), while MP38 was found in a VL case from the Reggio Emilia province and in an infected female sand fly from the Bologna province (S1 Table).

Half of the repeated MPs were obtained in the same year, while the other half were detected over a time interval of 1 year (MP8, MP19, MP23) or 2 years (MP38) (S1 Table).

### Population structure of *L*. *infantum* in the E-R region

**Fig 2:**
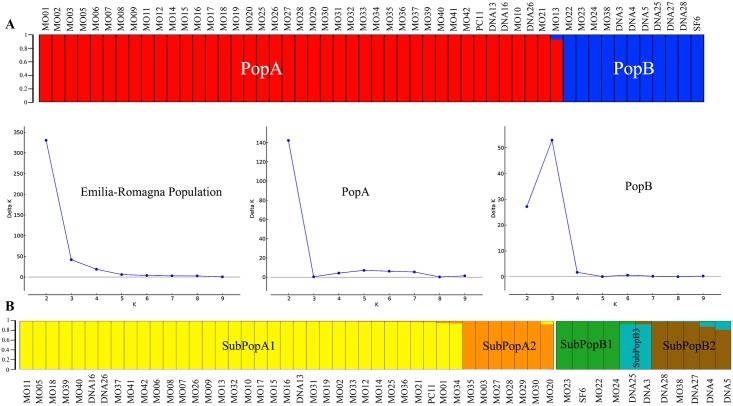
Estimated population structure for the 52 *L*. *infantum* strains from the Emilia-Romagna region (northeastern Italy), as inferred by STRUCTURE software on the basis of data for 15 microsatellite markers. Each strain is represented by a single vertical line divided into K colors, where K is the number of populations that were assumed. Each color represents one population, and the length of the colored segment shows the strain’s estimated proportion of membership (Q) in that population. **(A)** The derived graph for ΔK shows a peak at K = 2, indicating the existence of two populations in the investigated strain set. **(B)** The derived graphs for ΔK indicated the existence of two subpopulations within PopA and three subpopulations within PopB.

**Fig 3:**
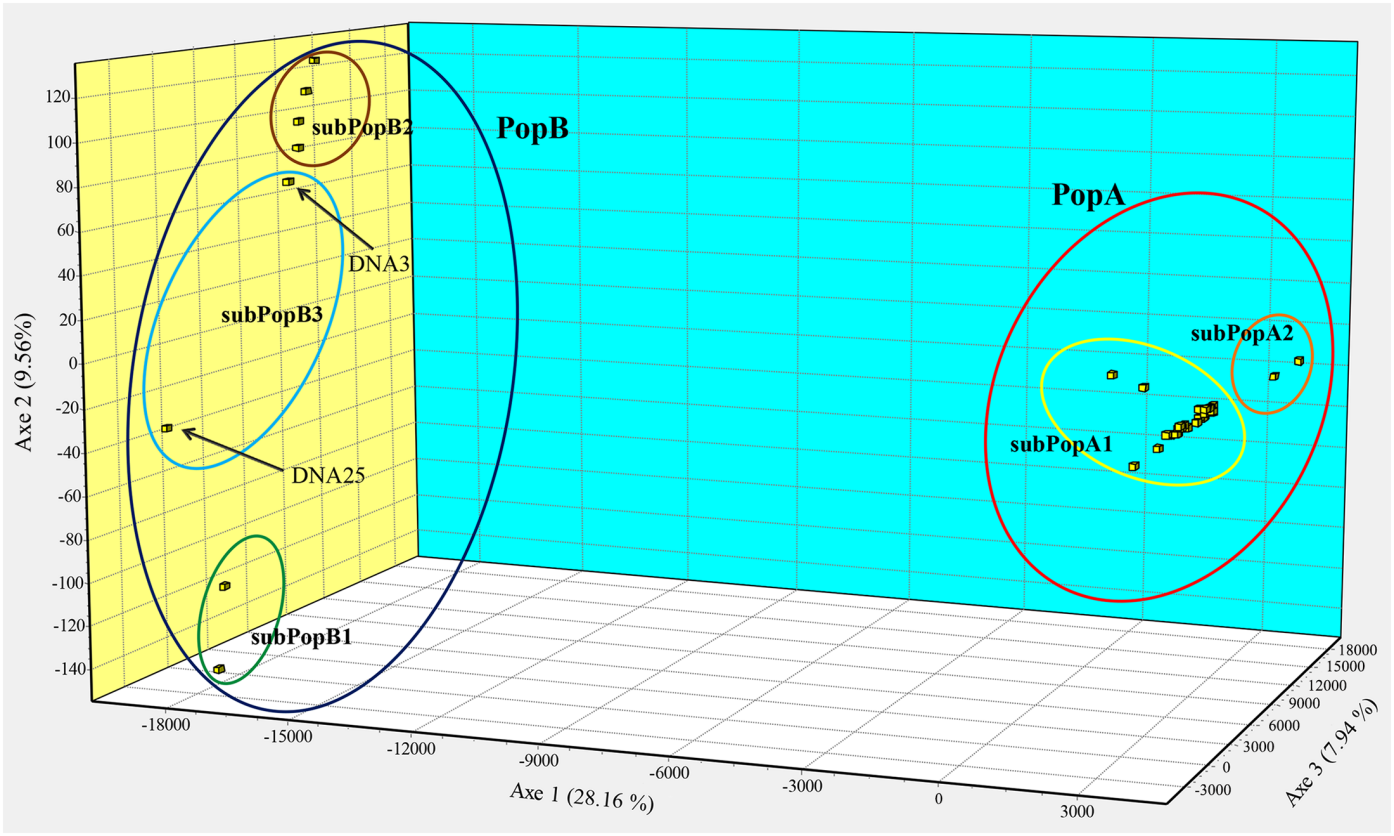
Spatial distribution of 52 *L*. *infantum* strains from the Emilia-Romagna region (northeastern Italy) by factorial correspondence analysis (FCA). Each square represents one microsatellite profile. The designations PopA (red circle), PopB (blue circle), subPopA1 (yellow circle), subPopA2 (orange circle), subPopB1 (green circle), subPopB2 (brown circle) and subPopB3 (light blue circle) correspond to the populations and subpopulations defined by STRUCTURE analysis as shown in Fig 2.

Samples obtained by pooled sand flies (n = 3) were excluded from population structure analysis. Bayesian clustering as implemented in STRUCTURE and calculation of ΔK values assigned the 52 *L*. *infantum* strains of the “E-R” subset to two main populations (Fig 2A); population A (PopA) consisted of 41 samples, including one VL case (DNA26) and 40 out of 40 canine samples. The second population (PopB) comprised 10 VL samples and the isolate obtained from one female of *Ph*. *perfiliewi*. Since the ΔK graph plot indicated further subdivision, the major populations were separately re-analyzed by STRUCTURE. This analysis revealed the existence of two clusters in PopA and three clusters in the PopB that were named subPopA1/subPopA2 and subPopB1/subPopB2/subPopB3, respectively (Fig 2B). The graphical representation of the factorial correspondence analysis (FCA) confirmed the assignment of the E-R samples to different genetic clusters, with emphasis of the distinct genetic isolation of populations A and B (Fig 3). On the other hand, members of subPopB3, DNA3 and DNA25, did not form a clear cluster in this analysis.

In addition, a phylogenetic tree was constructed from the MLMT data (S1 Fig). The obtained tree showed two well supported branches corresponding to the populations PopA and PopB as inferred by STRUCTURE analysis. With the exception of subPopB3, all the subpopulations were confirmed by the topology of the phylogenetic tree. Subsequently, measures for genetic differentiation were calculated for the *L*. *infantum* populations and subpopulations from the E-R region estimated by STRUCTURE (Table 3). F_st_ statistics revealed a strong genetic differentiation (>0.25) between PopA and PopB. Accordingly, a low migration rate was observed between these two main groups (Nm = 0.12). Similar results were observed in each pairwise comparison between subpopulations.

**Table 3:** Differentiation measures (F_st_), probability (*p*-value) and migration rate (Nm) between populations/subpopulations of *L*. *infantum* strains (n = 52) from the Emilia-Romagna region (northeastern Italy) as assumed by STRUCTURE.

	F_st_	*p*-value	Nm
PopA *vs* PopB	0.681	0.0001	0.12
subPopA1 *vs* subPopA2	0.501	0.0006	0.24
subPopB1 *vs* subPopB2	0.824	0.0064	0.05
subPopB1 *vs* subPopB3	0.547	0.0361	0.21
subPopB2 vs subPopB3	0.409	0.0298	0.36

Descriptive analysis per population and subpopulation was also performed (Table 4). A deficit of heterozygosity was observed for all genetic groups, as showed by high F_is_ values (>0.500). The mean expected heterozygosity (H_e_) values, as a measure of genetic diversity, were low to moderate within the 5 subpopulations, ranging from 0.018 to 0.333. SubPopA1 and subPopB3 were found to be the most variable sub-groups by having the highest MNA and H_e_ values (Table 4).

**Table 4:** Population genetic characterization of the populations and subpopulations found for 52 *L*. *infantum* strains from the Emilia-Romagna region (northeastern Italy).

Population	N	P	MNA	*H*_*e*_	*H*_*o*_	*F*_*is*_
PopA	41	0.667	3.000	0.259	0.068	0.738
PopB	11	0.667	1.800	0.269	0.042	0.848
**Subpopulation**						
subPopA1	34	0.667	3.000	0.234	0.082	0.651
subPopA2	7	0.067	1.067	0.018	0.000	1.000
subPopB1	4	0.067	1.067	0.038	0.000	1.000
subPopB2	5	0.267	1.267	0.081	0.040	0.538
subPopB3	2	0.533	1.533	0.333	0.133	0.692

Population/subpopulation, cluster as inferred by STRUCTURE analysis; N, sample size; P, proportion of polymorphic loci; MNA, mean number of alleles; He, expected heterozygosity; Ho, observed heterozygosity; F_is_, inbreeding coefficient

The geographical distribution of the different strains, corresponding to the genetic groups identified by Bayesian analysis, is shown in Fig 1. The assignment of samples to populations/subpopulations did not correspond to their geographical origin, with the exception of subPopA2 that was composed of 7 strains from dogs of the eastern area of the E-R region. Moreover, samples from the E-R region split among different populations/subpopulation with no correlation to year of collection.

### Comparative analysis between “E-R” and “extra E-R” subsets

Microsatellite analysis of the “extra E-R” subset (n = 10) detected 10 distinct MPs of which MP13 (Liguria region, northwestern Italy) was shared with a canine sample (MO31) from E-R.

STRUCTURE analysis (ΔK; K = 2) performed on all 62 Italian strains assigned all the “extra-E-R” samples to PopA, with the exception of a VL sample (PV4) from southern Italy, which showed partial membership in PopB (Q-PopA = 0.751; Q-PopB = 0.249) (S2 Fig). Population characteristics after merging “E-R” and “extra E-R” subsets are shown in S3 Table. The genetic diversity (H_e_) of PopA showed an increase from 0.259 to 0.273, and an increase in the mean number of alleles from 3.00 to 3.47 was observed. Samples from both subsets (E-R and extra E-R) within PopA shared most of the alleles. On the other hand, a total of 40 and 17 private alleles were found to be specific for PopA and PopB, respectively (S3 Table).

### Phylogenetic inference of the study strains and other selected strains belonging to the *L*. *donovani* complex

**Fig 4:**
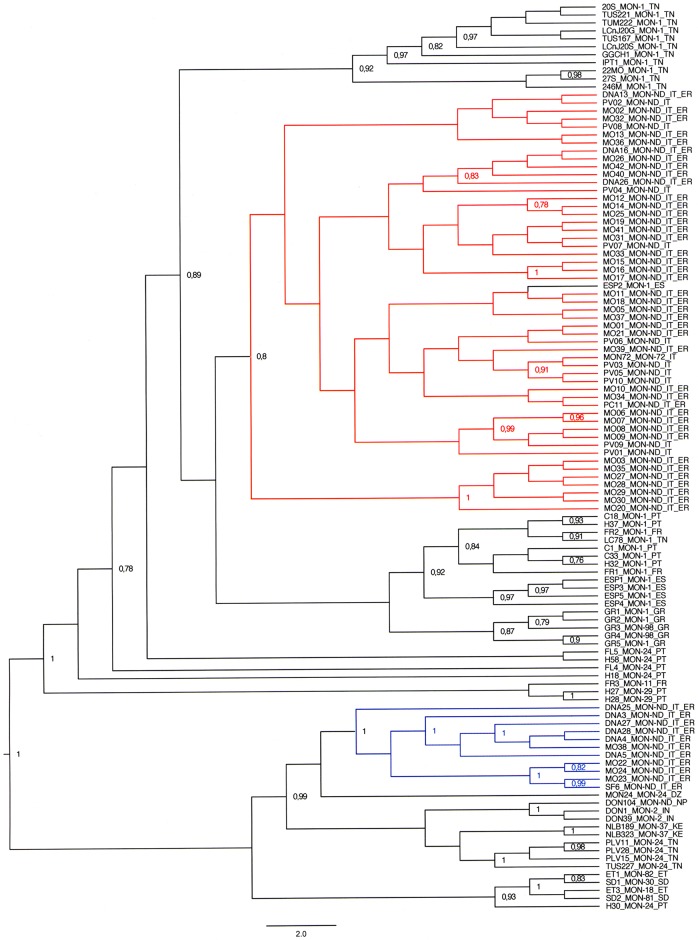
Bayesian phylogenetic tree (cladogram) obtained by the Sainudiin model [29] using data of 14 coincident microsatellite loci for the 62 *L*. *infantum* strains from Italy, 49 MLMT profiles available in literature and 3 WHO reference strains. Posterior probabilities > 0.75 are showed near the nodes. Strains representing the different microsatellite profiles are listed in S1 and S2 Tables. Strains designations specify, respectively, the laboratory code, the zymodeme (ND, not defined), the geographical origin (IT, Italy; ER, Emilia-Romagna; FR, France; SP, Spain; GR, Greece; PT, Portugal; DZ, Algeria; TN, Tunisia; KE, Kenia; SD, Sudan; ET, Ethiopia; IN, India; NP, Nepal). Italian samples are differentiated by the color of STRUCTURE designation (K = 2): PopA samples in red, PopB samples in blue.

A phylogenetic tree for 14 coincident markers was created including the 62 MLMT profiles obtained in this study, 49 selected strains of the *L*. *donovani* complex [16–19] (S2 Table) and 3 WHO reference strains (Fig 4). Italian strains belonging to PopA were grouped in a monophyletic clade together with the European and North African strains belonging to the zymodeme MON-1; this cluster also included the MON-98 strains from Greece and the MON-72 WHO reference strain. Samples of the “extra E-R” subset were intermingled with canine samples of E-R, confirming the close relatedness observed by STRUCTURE analysis.

On the other hand, Italian PopB samples gathered in a monophyletic clade that was more closely related to *L*. *infantum* MON-24 strains from North Africa (i.e. Algeria and Tunisia) and to *L*. *donovani* strains than to the other *L*. *infantum* strains.

The *L*. *infantum* MON-1 clade was divided in sub-clades according to the geographic origin of the examined strains. Exceptions were represented by the Spanish strain ESP2 (MHOM/ES/86/BCN16) and the strain LC78 (MHOM/TN/2004/LC78) from Tunisia, which clustered with strains from Italy and France/Portugal, respectively.

## Discussion

In this study, population genetics of *L*. *infantum* strains from northeastern Italy was investigated by applying MLMT analysis. Considerable genetic variation was disclosed for 55 *L*. *infantum* strains from the E-R region, which exhibited 41 different microsatellite profiles, thus leading to a genotype frequency of 0.050. Similar high genetic variability was reported for *L*. *infantum* populations from other Mediterranean countries, such as Portugal (0.043), Tunisia (0.059), Morocco (0.071), Algeria (0.071) and Greece (0.037) [18,19,35–37].

All the population genetic analyses employed in this study exposed two well defined populations among the 52 *L*. *infantum* samples from the E-R region; all CanL samples belonged to PopA, while all but one VL samples and a sand fly sample were part of a separated population, named PopB. The great differentiation of these genetic groups was supported by the high F_st_ value and by the presence of population-specific alleles. Even though pooled sand flies were excluded from clustering analysis, these samples exhibited an allelic composition typical for PopB.

PopA and PopB overlap geographically, as showed in Fig 1, while exhibiting a host-related genetic structure. In agreement with previous MLMT studies that did not detect any correlation between distinct genotypes and the host background [14,19,35,36], a close genetic relationship was observed in this study between CanL samples from the E-R region and VL samples from other Italian regions. Conversely, all but one human samples from the E-R region segregated separately in PopB. It is believed that *Leishmania* strains can exhibit different growth behavior in culture media [11]. Nevertheless, it seems unlikely that the microsatellite differences observed between E-R canine and human samples could be due to genotype selection during culture, as genetic differentiation was independent by the type of sample, i.e. isolate *versus* biological specimen. Interestingly, biological samples from dogs and humans exhibited homozygosis for population-specific alleles, with no evidence of infection sustained by mixed *L*. *infantum* populations.

A similar epidemiological pattern was observed with previous analysis of three genetic markers, i.e. cysteine protease B (*cpb*) gene, *k26*-gene and a repetitive nuclear region on chromosome 31 that were evaluated in *Leishmania* strains from the E-R region, showing circulation of distinct genotypes between dogs and humans that matched with the genetic groups observed in this study [9]. Since *k26* and *cpb* are protein-coding genes involved in the host/parasite and vector/parasite interaction [38,39], the two *L*. *infantum* populations circulating in the region could exhibit different phenotypic characteristics, which deserves further investigations.

The phylogenetic reconstruction of strains within the *L*. *donovani* complex grouped the PopA samples in a monophyletic clade that includes all the Mediterranean MON-1 strains as well as strains closely related to the MON-1 zymodeme (i.e. MON-98, MON-72) [14,40]. A large part of PopA samples has been previously characterized by *k26*-typing and exhibits an amplicon size of 626 bp, which is typical for the MON-1 zymodeme [9], thus further supporting the MLMT results. On the other hand, the E-R samples belonging to PopB exhibited great genetic distance from the MON-1 clade; interestingly, these samples were assigned to a distinct genetic group clearly separated from both European MON-1 and non MON-1 strains, and close to MON-24 strains from Tunisia and Algeria.

Therefore, the split observed at the uppermost hierarchical level (K = 2) between canine and human samples from the E-R region corresponds to the differentiation among MON-1 and non MON-1 groups, respectively, which is consistent with previous MLMT analysis of *L*. *infantum* strains [12,14]. This finding could explain the different host-specificity exhibited by PopA and PopB. In fact, *L*. *infantum* MON-1 zymodeme and zymodemes closely related (i.e. MON-77, MON-98, MON-108) [11] could have better fitness for the canine host, while non MON-1 zymodemes have been only occasionally isolated from dogs [14] and their reservoir hosts remain unknown.

Further clustering analysis indicated hierarchical genetic structure among *L*. *infantum* samples from the E-R region; two subpopulations (i.e. subPopA1/A2) and three sub-populations (i.e. SubPopB1/B2/B3) were exposed within PopA and PopB, respectively. However, subpopulation B3 was not well supported by FCA and phylogenetic analysis. Samples included in this cluster (DNA3 and DNA25) were from two VL patients co-infected with Human Immunodeficiency Virus. Interestingly, DNA25 showed bi-allelic loci with alleles characteristics for subPopB1 and subPopB2. We cannot exclude that heterozygous alleles are due to recombination or mixed infection.

Subpopulations only partially matched with their geographic origin (Fig 1); however, this study was carried out in a relatively small area of northeastern Italy, without physical geographic barriers to the parasite flow, as evidenced by the isolation of strains with same MPs in different provinces of the E-R region. Interestingly, MP38 was shared by a sand fly sample and by a VL case occurring in a 30 Km area. Given the typically short flight-range of sand flies [41], factors other than vector movement are likely involved in parasite distribution over that geographic distance, such as movements of infected individuals or animal reservoirs. Within PopA, subPopA1 was widespread over the region and showed the highest levels of genetic diversity and allelic richness. In addition, microsatellite patterns appeared to be quite stable over time, as evidenced by the phylogenetic position of the oldest sample available (PC11) that was collected in 1998. This subpopulation might have been introduced from other Italian regions due to active reservoir migration, as previously hypothesized for the reported northward expansion of *L*. *infantum* in Italy [3]. However, the small number of extra E-R samples and their wide geographical dispersal did not allow to identify the origin of parasite migration. The cluster subPopA2, that was restricted to the eastern part of E-R, could be related to a different transmission cycle or could have spread in this area from other endemic regions not covered in this study.

As a hypothesis to explain the presence of two sympatric populations (PopA and PopB), with weak genetic exchange, two overlapping transmission cycles could occur in the studied area, involving different sand fly vectors and/or reservoirs.

The sand fly fauna of the E-R region presents an overwhelming abundance of *Phlebotomus* (*Ph*.) *perfiliewi* (98%) and a low percentage of *Ph*. *perniciosus* (2%) [9,42], the latter being more abundant in the rest of Italy and considered the most efficient vector of *L*. *infantum* in the country [3,43]. Nevertheless, the role of *Ph*. *perfiliewi* as a vector of dermotropic *L*. *infantum* strains has been reported in the Abruzzo region (central Italy) [44] and it has been suggested in Tunisia [45].

We hypothesize that the peculiar composition of the sand fly fauna in the E-R region may play a role in the selection of particular *Leishmania* strains, such as PopB, that was demonstrated to circulate in humans and vectors. A role of sand flies in shaping the genetic variability of *L*. *infantum* populations has been previously suggested by Ferreira et al. [46]. In support to our hypothesis, the leishmanial isolate obtained from one female of *Ph*. *perfiliewi* that was captured in the E-R region belonged to PopB. Notably, no leishmanial strains responsible for CanL transmission (i.e. PopA) have been found so far in sand flies. Therefore, further studies are needed to assess whether *Ph*. *perfiliewi* and *Ph*. *perniciosus* exhibit distinct vectorial competence for different leishmanial strains. Moreover, a comprehensive entomological study as recently performed by Alten and colleagues [43] would be needed to provide patterns of the potential behavior of leishmaniasis vectors.

Dog is considered the main reservoir host of *L*. *infantum* as well as the main source of human infection [2]. Nevertheless, seroprevalence of *L*. *infantum* in the canine population from the E-R region is low when compared to the CanL seroprevalence of other Italian endemic areas (2% vs 18%, respectively) [47], and it shows a decreasing trend over time [8]. Conversely, increasing numbers of human VL cases were reported in two provinces of the E-R region (Modena and Bologna) in recent years [6,7]. This observation, together with the previous [9] and current findings on genetic isolation between CanL and VL strains from the E-R region raise questions on the likelihood of dogs as the main reservoirs for VL in this area. Thus, the potential role of other mammals, including lagomorphs, wild and domestic carnivores (i.e. foxes, cats), and rodents should be extensively examined in the E-R region.

In conclusion, this study showed genetic structuring within *L*. *infantum* population from the E-R region, separating the strains into two sympatric groups that exhibit different host specificity. A comprehensive surveillance monitoring parasitic strains obtained from vectors, human and non-human hosts, is warranted to elucidate the hypothesized overlapping epidemiological cycles and to improve control measures for leishmaniasis in this area.

## Supporting information

S1 Fig Bayesian phylogenetic tree obtained by the Sainudiin model [29] using data of 15 microsatellite loci for the 52 *L*. *infantum* strains from the Emilia-Romagna region (northeastern Italy). Posterior probability values >0.75 are indicated at the nodes. Strains representing the different microsatellite profiles are listed in S1 Table. Populations and sub-populations as inferred by STRUCTURE are indicated by colored bars: red for PopA, blue for PopB, yellow for subPopA1; orange for subPopA2; green for subPopB1; brown for subPopB2; light blue for subPopB3.
(TIF)

S2 Fig Estimated population structure for *L*. *infantum* from Italy as inferred by STRUCTURE software on the basis of data for 15 microsatellite markers obtained for the 62 strains (52 from the Emilia Romagna region and 10 extra regional). Extra E-R strains are presented in bold. Each strain is represented by a single vertical line divided into K colors, where K is the number of populations assumed. Each color represents one population and the length of the colored segment shows the strain’s estimated proportion of membership (Q) in the specific population. The derived graph for ΔK shows a major peak at K = 2, indicating the presence of two populations in the investigated sample set.
(TIF)

S1 Table Designation, characteristics and MLMT profiles of the *Leishmania*-positive samples and reference strains included in the study. Fragment sizes of each marker were obtained by multiplying the normalized repeat numbers by two (dinucleotide microsatellites were used) and adding the size of the flanking regions. Grey-filled rows indicate samples with repeated microsatellite profiles. WHO, World Health Organization; n.d., not defined; CanL, Canine leishmaniasis; VL, Visceral leishmaniasis; HIV+, Human Immunodeficiency Virus Positive (N, no; Y, yes); E-R, Emilia-Romagna region; PC, Piacenza province; PR, Parma province; RE, Reggio Emilia province; MO, Modena province; BO, Bologna province; RA, Ravenna province; FC, Forlì-Cesena province; RN, Rimini province; GE, Genova province; PV, Pavia province; n.a., null allele; Pop, population; subPop, subpopulation. *, this locus contains the mutation TG/CG in 9^th^ position, which was arbitrarily included in the repeat number; ^, this locus exhibits same length but one repeat (CA) more when compared with the strain MHOM/ES/93/PM1 (GenBank accession number: AM050049), which was used as size reference for typing *L*. *infantum* strains listed in S2 Table.
(XLSX)

S2 Table *Leishmania donovani* complex strains used in this study for comparison. Nd, not done.
(DOCX)

S3 Table Characteristics of the 15 microsatellite markers used for population analysis of Italian *Leishmania infantum* strains (N = 62). Pop, population; N, sample size; A, number of alleles; He, expected heterozygosity; Ho, observed heterozygosity; F_is_, inbreeding coefficient. In brackets the respective values obtained including the “extra E-R” samples (N = 10) are given. Predominating alleles are marked as bold numbers.
(DOCX)

S1 Protocol Protocol and primer pairs used for sequencing the 15 microsatellite loci of the strain MHOM/TN/1980/IPT1, used as standard for fragments’ size in each MLMT run. (DOCX)

## References

[1] World Health Organization (WHO). Control of the leishmaniases: report of a meeting of the WHO Expert Committee on the Control of Leishmaniases, Geneva, 22–26 March 2010. http://apps.who.int/iris/handle/10665/44412.

[2] Alvar J, Vélez ID, Bern C, Herrero M, Desjeux P, Cano J, et al Leishmaniasis Worldwide and global estimates of its incidence. PLoS ONE. 2012; 7(5): e35671 10.1371/journal.pone.0035671 . 22693548 PMC3365071

[3] Maroli M, Rossi L, Baldelli R, Capelli G, Ferroglio E, Genchi C, et al The northward spread of leishmaniasis in Italy: evidence from retrospective and ongoing studies on the canine reservoir and phlebotomine vectors. Trop Med Int Health 2008; 13(2): 256–64. 10.1111/j.1365-3156.2007.01998.x . 18304273

[4] Biglino A, Bolla C, Concialdi E, Trisciuoglio A, Romano A, Ferroglio E. Asymptomatic *Leishmania infantum* infection in an area of northwestern Italy (Piedmont region) where such infections are traditionally nonendemic. J Clin Microbiol. 2010; 48: 131–136. 10.1128/JCM.00416-09 . 19923480 PMC2812267

[5] Gramiccia M, Scalone A, Di Muccio T, Orsini S, Fiorentino E, Gradoni L. The burden of visceral leishmaniasis in Italy from 1982 to 2012: a retrospective analysis of the multi-annual epidemic that occurred from 1989 to 2009. Euro Surveill. 2013;18(29):20535 . 23929120

[6] Varani S, Cagarelli R, Melchionda F, Attard L, Salvadori C, Finarelli AC, et al Ongoing outbreak of visceral leishmaniasis in Bologna Province, Italy, November 2012 to May 2013. Euro Surveill 2013; 18:20530 . 23929116

[7] Franceschini E, Puzzolante C, Menozzi M, Rossi L, Bedini A, Orlando G, et al Clinical and microbiological characteristics of visceral leishmaniasis outbreak in a northern italian nonendemic area: a restrospective observational study. Biomed Res Int. 2016; 10.1155/2016/6481028 . 27999807 PMC5141564

[8] Santi A, Renzi M, Baldelli R, Calzolari M, Caminiti A, Dell’Anna S, et al A surveillance program on canine leishmaniasis in the public kennels of Emilia-Romagna Region, Northern Italy. Vector Borne Zoonotic Dis 2014; 14: 206‒211. 10.1089/vbz.2013.1362 . 24575787 PMC3952590

[9] Rugna G, Carra E, Corpus F, Calzolari M, Salvatore D, Bellini R, et al Distinct *Leishmania infantum* strains circulate in humans and dogs in the Emilia-Romagna Region, northeastern Italy. Vector Borne Zoonotic Dis 2017; 17: 409–415. 10.1089/vbz.2016.2052 . 28301296

[10] Schönian G, Mauricio IL, Gramiccia M, Cañavate C, Boelaert M, Dujarden JC. Leishmaniases in the Mediterranean in the era of molecular epidemiology. Trends Parasitol. 2008; 24(3): 135‒142. 10.1016/j.pt.2007.12.006 . 18262469

[11] Schönian G, Kuhls K, Mauricio IL. Molecular approaches for a better understanding of the epidemiology and population genetics of *Leishmania*. Parasitology 2011; 138(4): 405‒25. 10.1017/S0031182010001538 . 21078222

[12] Ochsenreither S, Kuhls K, Schaar M, Presber W, Schönian G. Multilocus microsatellite typing as a new tool for discrimination of *Leishmania infantum* MON-1 strains. J Clin Microbiol. 2006;44: 495–503. 10.1128/JCM.44.2.495-503.2006 . 16455904 PMC1392658

[13] Kuhls K, Keilonat L, Ochsenreither S, Schaar M, Schweynoch C, Presber W, et al Multilocus microsatellite typing (MLMT) reveals genetically isolated populations between and within the main endemic regions of visceral leishmaniasis. Microbes Infect. 2007; 9: 334–343. 10.1016/j.micinf.2006.12.009 . 17307010

[14] Kuhls K, Chicharro C, Cañavate C, Cortes S, Campino L, Haralambous C, et al Differentiation and gene flow among European populations of *Leishmania infantum* MON-1. PLoS Negl Trop Dis. 2008; 2(7): e261 10.1371/journal.pntd.0000261 . 18612461 PMC2438616

[15] Jamjoom MB, Ashford RW, Bates PA, Kemp SJ, Noyes HA. Towards a standard battery of microsatellite markers for the analysis of the *Leishmania donovani* complex. Ann Trop Med Parasitol. 2002; 96: 265–270. 10.1179/000349802125000790 . 12061973

[16] Alam MZ, Kuhls K, Schweynoch C, Sundar S, Rijal S, Shamsuzzaman AKM, et al Multilocus microsatellite typing (MLMT) reveals genetic homogeneity of *Leishmania donovani* strains in the Indian subcontinent. Infect Genet Evol J Mol Epidemiol Evol Genet Infect Dis. 2009; 9(1): 24–31. 10.1016/j.meegid.2008.09.005 . 18957333

[17] Alam MZ, Haralambous C, Kuhls K, Gouzelou E, Sgouras D, Soteriadou K, et al The paraphyletic composition of *Leishmania donovani* zymodeme MON-37 revealed by multilocus microsatellite typing. Microbes Infect. 2009; 11(6–7): 707–715. 10.1016/j.micinf.2009.04.009 . 19376262

[18] Cortes S, Maurício IL, Kuhls K, Nunes M, Lopes C, Marcos M, et al Genetic diversity evaluation on Portuguese *Leishmania infantum* strains by multilocus microsatellite typing. Infect Genet Evol J Mol Epidemiol Evol Genet Infect Dis. 2014; 26: 20–31. 10.1016/j.meegid.2014.04.023 . 24815728

[19] Chargui N, Amro A, Haouas N, Schönian G, Babba H, Schmidt S, et al Population structure of Tunisian *Leishmania infantum* and evidence for the existence of hybrids and gene flow between genetically different populations. International Journal for Parasitology 2009; 39: 801–811. 10.1016/j.ijpara.2008.11.016 . 19211023

[20] Gramiccia M. The identification and variability of the parasites causing leishmaniasis in HIV-positive patients in Italy. Ann Trop Med Parasitol. 2003; 97 (1):65–73. 10.1179/000349803225002543 . 14678634

[21] El Tai NO, El Fari M, Mauricio I, Miles MA, Oskam L, El Safi SH, Presber WH, Schönian G. *Leishmania donovani*: intraspecific polymorphisms of Sudanese isolates revealed by PCR-based analyses and DNA sequencing. Exp Parasitol. 2001; 97(1):35–44. 10.1006/expr.2001.4592 . 11207112

[22] Reale S, Lupo T, Migliazzo A, Di Mauro C, Cipri V, Calderone S, et al Multilocus microsatellite polymorphism analysis to characterize *Leishmania infantum* strains isolated in Sicily. Transbound Emerg Dis 2010; 57: 37–41. 10.1111/j.1865-1682.2010.01131.x . 20537100

[23] Pritchard JK, Stephens M, Donnelly P. Inference of population structure using multilocus genotype data. Genetics 2000; 155, 945–959. . 10835412 10.1093/genetics/155.2.945 PMC1461096

[24] Evanno G, Regnaut S, Goudet J. Detecting the number of clusters of individuals using the software STRUCTURE: a simulation study. Mol Ecol 2005; 14: 2611–2620. 10.1111/j.1365-294X.2005.02553.x . 15969739

[25] Earl DA, Von Holdt BM. STRUCTURE HARVESTER: a website and program for visualizing STRUCTURE output and implementing the Evanno method. Conserv Genet Resour 2012; 4: 359–361.

[26] Jakobsson M, Rosenberg NA. CLUMPP: a cluster matching and permutation program for dealing with label switching and multimodality in analysis of population structure. Bioinformatics. 2007; 23: 1801–1806. 10.1093/bioinformatics/btm233 . 17485429

[27] Ramasami RK, Ramasami S, Bindroo BB, Naik G. STRUCTURE PLOT: a program for drawing elegant STRUCTURE bar plots in user friendly interface. SpringerPlu 2014; 3: 431 10.1186/2193-1801-3-431 . 25152854 PMC4141070

[28] Belkhir K, Borsa P, Chikhi L, Raufastey N, Bonhomme F. (2004) GENETIX 4.05, logiciel sous Windows TM pour la génétique des populations. Laboratoire Génome, Populations, Interactions, CNRS UMR 5000, Université de Montpellier II, Montpellier (France).

[29] Sainudiin R, Durrett RT, Aquadro CF, Nielsen R. Microsatellite mutation models: insights from a comparison of humans and chimpazees. Genetics 2004; 168(1): 383–395. 10.1534/genetics.103.022665 . 15454551 PMC1448085

[30] Bouckaert R, Heled J, Kühnert D, Vaughan T, Wu C-H, Xie D, et al BEAST 2: A Software Platform for Bayesian Evolutionary Analysis. PLoS Computational Biology 2014; 10(4): e1003537 10.1371/journal.pcbi.1003537 . 24722319 PMC3985171

[31] Lewis P, Zaykin D. Genetic data analysis: computer programme for the analysis of allelic data. 2001 Version 1.0 (d16c). Free program distributed by the authors over the internet from http://lewis.eeb.uconn.edu/lewishome/software.html.

[32] Weir BS, Cockerham CC. Estimating F statistics for the analysis of population structure. Evolution 1984; 38, 1358–1370. 10.1111/j.1558-5646.1984.tb05657.x . 28563791

[33] Dieringer D, Schlötterer C. Microsatellite analyser (MSA): a platform independent analysis tool for large microsatellite data sets. Molecular Ecology Notes 2003; 3: 167–169.

[34] Wright S. Evolution and the genetics of populations Variability within and among Natural Populations, vol. 4 The University of Chicago Press, Chicago, 1978.

[35] Amro A, Hamdi S, Lemrani M, Mouna I, Mohammed H, Mostafa S, et al Moroccan *Leishmania infantum*: genetic diversity and population structure as revealed by Multi-Locus Microsatellite Typing. PLoS ONE. 2013; 8(10): e77778 10.1371/journal.pone.0077778 . 24147078 PMC3798341

[36] Seridi N, Amro A, Kuhls K, Belkaid M, Zidane C, Al-Jawabreh A, et al Genetic polymorphism of Algerian *Leishmania infantum* strains revealed by multilocus microsatellite analysis. Mirobes Infect. 2008; 10(12–13): 1309–1315. 10.1016/j.micinf.2008.07.031 . 18755285

[37] Gouzelou E, Haralambous C, Antoniou M, Christodoulou V, Martinković F, Živičnjak T et al Genetic diversity and structure in *Leishmania infantum* populations from southeastern Europe revealed by microsatellite analysis. Parasit Vectors. 2013; 6:342 10.1186/1756-3305-6-342 . 24308691 PMC4029556

[38] Hide M, Bras-Gonçalves R, Bañuls AL. Specific *cpb* copies within the *Leishmania donovani* complex: evolutionary interpretations and potential clinical implications in humans. Parasitology 2007; 134(3): 379–389. 10.1017/S0031182006001600 . 17129395

[39] Zackay A, Nasereddin A, Takele Y, Tadesse D, Hailu W, Hurissa Z, et al Polymorphism in the HASPB Repeat Region of East African *Leishmania donovani* Strains. PLoS Negl Trop Dis 2013; 7(1): e2031 10.1371/journal.pntd.00020 23358849 PMC3554577

[40] Amro A, Schönian G, Al-Sharabati MB, Azmi K, Nasereddin A, Abdeen Z, et al Population genetics of *Leishmania infantum* in Israel and Palestinian Authority through microsatellite analysis. Microbes Infect. 2009; 11(4): 484–492. . 19399967 10.1016/j.micinf.2009.02.001

[41] Maroli M, Feliciangeli MD, Bichaud L, Charrel RN, Gradoni L. Phlebotomine sandflies and the spreading of leishmaniases and other diseases of public health concern. Medical and veterinary entomology, 2012 10.1111/j.1365-2915.2012.01034.x . 22924419

[42] Calzolari M, Angelini P, Finarelli AC, Cagarelli R, Bellini R, Albieri A, et al Human and entomological surveillance of Toscana virus in the Emilia-Romagna region, Italy, 2010 to 2012. Euro Surveill 2014; 19: 20978 . 25496572 10.2807/1560-7917.es2014.19.48.20978

[43] Alten B, Maia C, Afonso MO, Campino L, Jiménez M, González E, et al Seasonal dynamics of Phlebotomine sand fly species proven vectors of Mediterranean leishmaniasis caused by *Leishmania infantum*. PLoS Negl Trop Dis. 2016; 10(2): e0004458 10.1371/journal.pntd.0004458 . 26900688 PMC4762948

[44] Maroli M, Gramiccia M, Gradoni L. Natural infection of the sandfly *Phlebotomus perfiliewi* Parrot, 1930 with *Leishmania infantum* Nicolle, 1908 in a cutaneous leishmaniasis focus of the Abruzzi region (Italy). Trans R Soc Trop Med Hyg. 1987; 81: 596–598. . 3445341 10.1016/0035-9203(87)90420-2

[45] Kallel K, Haouas N, Pratlong F, Kaouech E, Belhadj S, Anane S, et al Cutaneous leishmaniasis caused by *Leishmania infantum* MON-24 in Tunisia: extension of the focus to the center of the country. Boll Soc Pathol Exot. 2008; 101(1): 29–31. . 18432004

[46] Ferreira GE, dos Santos BN, Dorval ME, Ramos TP, Porrozzi R, Peixoto AA, et al The genetic structure of *Leishmania infantum* populations in Brazil and its possible association with the transmission cycle of visceral leishmaniasis. PLoS ONE 2012; 7(5): e36242 10.1371/journal.pone.0036242 . 22606248 PMC3350531

[47] Maia C, Cardoso L. Spread of *Leishmania infantum* in Europe with dog travelling. Vet Parasitol. 2015; 213: 2–11. 10.1016/j.vetpar.2015.05.003 . 26021526

