# Supplementary figures and images for: Multilocus microsatellite typing (MLMT) reveals host-related population structure in Leishmania infantum from northeastern Italy
Source: PLoS Negl Trop Dis. 2018 Jul 5;12(7):e0006595. doi: 10.1371/journal.pntd.0006595 (PMC6057669; doi:10.1371/journal.pntd.0006595)

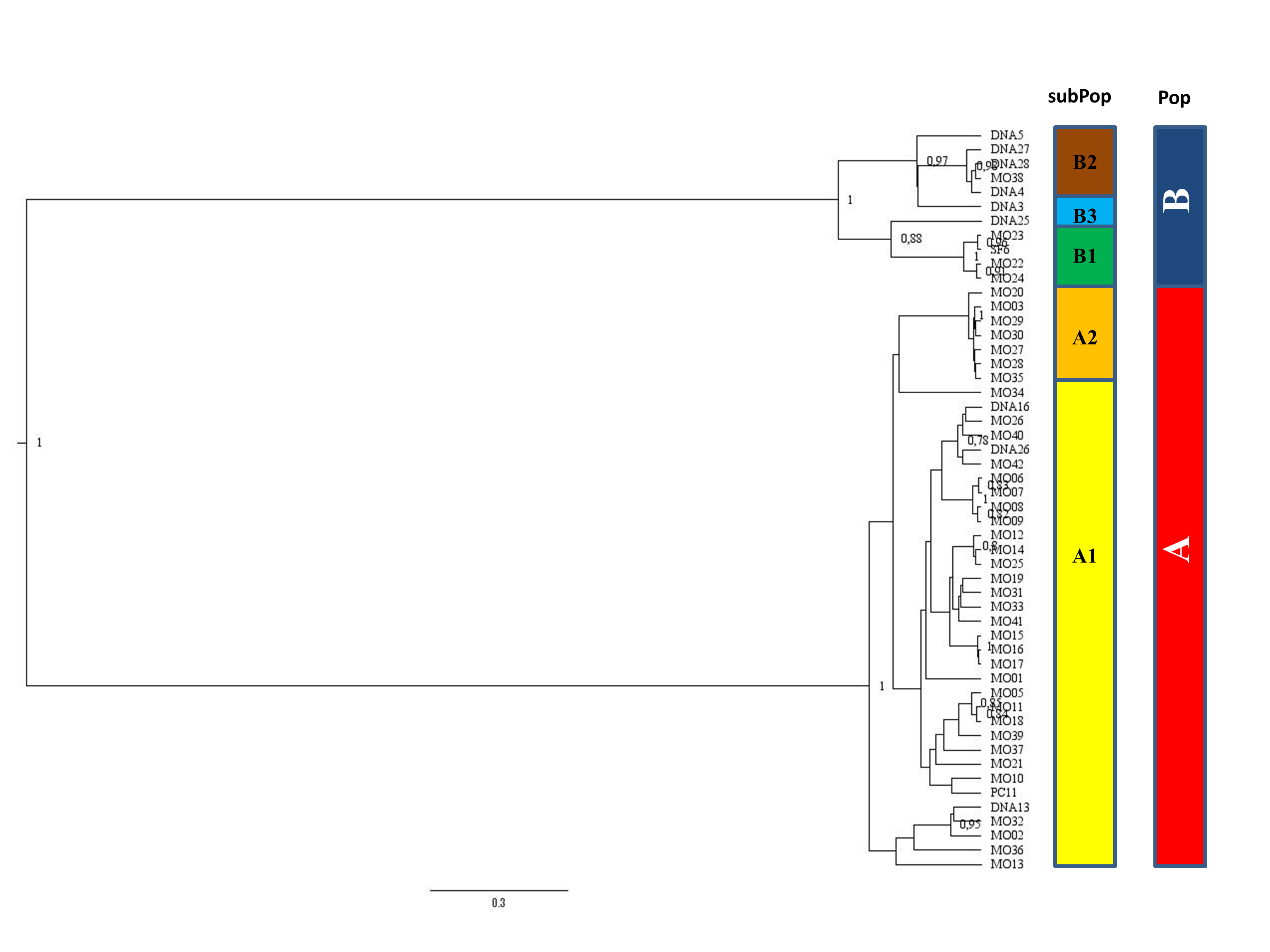

Supplement: S1 Fig — Posterior probability values >0.75 are indicated at the nodes. Strains representing the different microsatellite profiles are listed in S1 Table. Populations and sub-populations as inferred by STRUCTURE are indicated by colored bars: red for PopA, blue for PopB, yellow for subPopA1; orange for subPopA2; green for subPopB1; brown for subPopB2; light blue for subPopB3. (TIF) [file pntd.0006595.s001.tif]

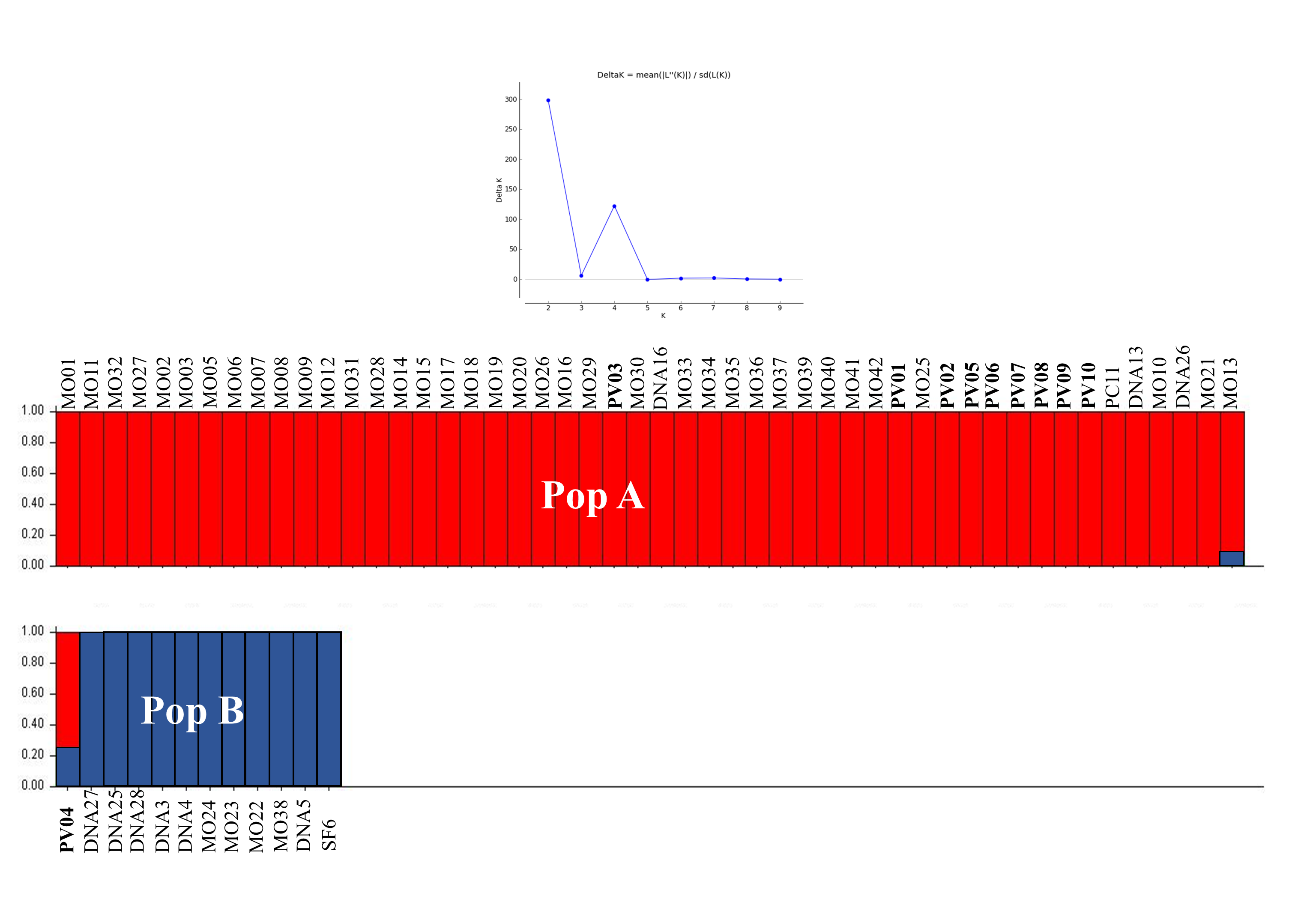

Supplement: S2 Fig — Extra E-R strains are presented in bold. Each strain is represented by a single vertical line divided into K colors, where K is the number of populations assumed. Each color represents one population and the length of the colored segment shows the strain’s estimated proportion of membership (Q) in the specific population. The derived graph for ΔK shows a major peak at K = 2, indicating the presence of two populations in the investigated sample set. (TIF) [file pntd.0006595.s002.tif]
