# Supplementary material for: Multilocus microsatellite typing (MLMT) reveals host-related population structure in Leishmania infantum from northeastern Italy
Source: PLoS Negl Trop Dis. 2018 Jul 5;12(7):e0006595. doi: 10.1371/journal.pntd.0006595 (PMC6057669; doi:10.1371/journal.pntd.0006595)
Supplement: S2 Table — Nd, not done. (DOCX) [file pntd.0006595.s004.docx]

| **Species** | **Lab code** | **Country** | **WHO-code** | **Zymodeme** | **Reference** |
| --- | --- | --- | --- | --- | --- |
| *Leishmania infantum* | H18 | Portugal | MHOM/PT/2004/IMT363 | MON-24 | [1] |
|  | H27 | Portugal | MHOM/PT/1997/IMT227 | MON-29 | [1] |
|  | H28 | Portugal | MHOM/PT/1997/IMT228 | MON-29 | [1] |
|  | H30 | Portugal | MHOM/PT/1992/IMT181 | MON-24 | [1] |
|  | H32 | Portugal | MHOM/PT/1989/IMT163 | MON-1 | [1] |
|  | H37 | Portugal | MHOM/PT/1993/IMT184 | MON-1 | [1] |
|  | H58 | Portugal | MHOM/PT/2004/IMT369 | MON-24 | [1] |
|  | C1 | Portugal | MCAN/PT/2003/IMT300 | MON-1 | [1] |
|  | C18 | Portugal | MCAN/PT/1995/IMT205 | MON-1 | [1] |
|  | C33 | Portugal | MCAN/PT/2003/IMT322 | MON-1 | [1] |
|  | FL4 | Portugal | IARI/PT/1989/IMT171 | MON-24 | [1] |
|  | FL5 | Portugal | IARI/PT/1989/IMT172 | MON-24 | [1] |
|  | ESP1 | Spain | MHOM/ES/93/PM1 | MON-1 | [1] |
|  | ESP2 | Spain | MHOM/ES/86/BCN16 | MON-1 | [1] |
|  | ESP3 | Spain | MHOM/ES/2001/LLM-981 | MON-1 | [1] |
|  | ESP4 | Spain | MCAN/ES/2001/LLM-1007 | MON-1 | [1] |
|  | ESP5 | Spain | MCAN/ES/2002/LLM-1139 | MON-1 | [1] |
|  | FR1 | France | MHOM/FR/97/LSL29 | MON-1 | [1] |
|  | FR2 | France | MHOM/FR/78/LEM75 | MON-1 | [1] |
|  | FR3 | France | MHOM/FR/80/LEM189 | MON-11 | [1] |
|  | GR1 | Greece | MCAN/GR/2002/GR9 | MON-1 | [1] |
|  | GR2 | Greece | MCAN/GR/2003/GR12 | MON-1 | [1] |
|  | GR3 | Greece | MHOM/GR/2003/GR19 | MON-98 | [1] |
|  | GR4 | Greece | MHOM/GR/2004/GR17 | MON-98 | [1] |
|  | GR5 | Greece | MHOM/GR/2002/GR26 | MON-1 | [1] |
|  | TUS227 | Tunisia | MHOM/TN/2002/Tus227 | MON-24 | [2] |
|  | PLV11 | Tunisia | MHOM/TN/2005/PLV11 | MON-24 | [2] |
|  | PLV15 | Tunisia | MHOM/TN/2005/PLV15 | MON-24 | [2] |
|  | PLV28 | Tunisia | MHOM/TN/2005/PLV28 | MON-24 | [2] |
|  | TUS167 | Tunisia | MHOM/TN/2001/Tus167 | MON-1 | [2] |
|  | 27S | Tunisia | MHOM/TN/2002/27M | MON-1 | [2] |
|  | 246M | Tunisia | MHOM/TN/2002/246M | MON-1 | [2] |
|  | 20S | Tunisia | MHOM/TN/2002/20S | MON-1 | [2] |
|  | 22MO | Tunisia | MHOM/TN/2002/22MO | MON-1 | [2] |
|  | TUS221 | Tunisia | MHOM/TN/2002/Tus221 | MON-1 | [2] |
|  | TUM222 | Tunisia | MHOM/TN/2002/Tum222 | MON-1 | [2] |
|  | LCnJ20S | Tunisia | MCAN/TN/2002/LCnJ20S | MON-1 | [2] |
|  | GGCH1 | Tunisia | MCAN/TN/2002/GGCH1/02 | MON-1 | [2] |
|  | LCnJ20G | Tunisia | MCAN/TN/2002/LCnJ20G | MON-1 | [2] |
|  | LC78 | Tunisia | MHOM/TN/2004/LC78 | MON-1 | [2] |
| *Leishmania donovani* | DON39 | India | MHOM/IN/0000/DEVI | MON-2 | [3] |
|  | DON1 | India | MHOM/IN/1980/DD8 | MON-2 | [3] |
|  | DON104 | Nepal | MHOM/NE/2003/BPK294/0 | nd | [3] |
|  | NLB189 | Kenia | MHOM/KE/1983/NLB189 | MON-37 | [4] |
|  | NLB323 | Kenia | MHOM/KE/1985/NLB323 | MON-37 | [4] |
|  | ET1 | Etiopia | MHOM/ET/72/GEBRE 1 | MON-82 | [1] |
|  | ET3 | Etiopia | MHOM/ET/67/LV9 | MON-18 | [1] |
|  | SD1 | Sudan | MHOM/SD/82/GILANI | MON-30 | [1] |
|  | SD2 | Sudan | MHOM/SD/62/3S | MON-81 | [1] |

**References**

1. Cortes S, Maurício IL, Kuhls K, Nunes M, Lopes C, Marcos M, et al. Genetic diversity evaluation on Portuguese Leishmania infantum strains by multilocus microsatellite typing. Infect Genet Evol J Mol Epidemiol Evol Genet Infect Dis. 2014; 26: 20–31. PMID: 24815728.
2. Chargui N, Amro A, Haouas N, Schönian G, Babba H, Schmidt S, et al. Population structure of Tunisian Leishmania infantum and evidence for the existence of hybrids and gene flow between genetically different populations. International Journal for Parasitology 2009; 39: 801–811. PMID: 19211023.
3. Alam MZ, Kuhls K, Schweynoch C, Sundar S, Rijal S, Shamsuzzaman AKM, et al. Multilocus microsatellite typing (MLMT) reveals genetic homogeneity of *Leishmania donovani* strains in the Indian subcontinent. Infect Genet Evol J Mol Epidemiol Evol Genet Infect Dis. 2009; 9(1): 24–31. PMID: 18957333.
4. Alam MZ, Haralambous C, Kuhls K, Gouzelou E, Sgouras D, Soteriadou K, et al. The Paraphyletic composition of *Leishmania donovani* zymodeme MON-37 revealed by multilocus microsatellite typing. Microbes Infect. 2009; 11(6-7): 707–715. PMID: 19376262.
