# Supplementary material for: Multilocus microsatellite typing (MLMT) reveals host-related population structure in Leishmania infantum from northeastern Italy
Source: PLoS Negl Trop Dis. 2018 Jul 5;12(7):e0006595. doi: 10.1371/journal.pntd.0006595 (PMC6057669; doi:10.1371/journal.pntd.0006595)
Supplement: S3 Table — Pop, population; N, sample size; A, number of alleles; He, expected heterozygosity; Ho, observed heterozygosity; Fis, inbreeding coefficient. In brackets the respective values obtained including the “extra E-R” samples (N = 10) are given. Predominating alleles are marked as bold numbers. (DOCX) [file pntd.0006595.s005.docx]

| **Marker** | **Population** | **Repeat array** | **Private allele** | **N** | **A** | **He** | **Ho** | **F_is_** |
| --- | --- | --- | --- | --- | --- | --- | --- | --- |
| Li41-56 | PopA | CA **10** |  | 41 (51) | 1 (1) | 0.000 (0.000) | 0.000 (0.000) | 0.000 (0.000) |
|  | PopB | CA **9 10** | 9 | 11 | 2 | 0.519 | 0.000 | 1.000 |
| Li46-67 | PopA | CA **10** | 10 | 41 (51) | 1 (1) | 0.000 (0.000) | 0.000 (0.000) | 0.000 (0.000) |
|  | PopB | CA **7** | 7 | 11 | 1 | 0.000 | 0.000 | 0.000 |
| Li21-34 | PopA | CA **15** 16 | 15 16 | 41 (51) | 2 (2) | 0.048 (0.039) | 0.000 (0.000) | 1.000 (1.000) |
|  | PopB | CA 8 **9** | 8 9 | 11 | 2 | 0.506 | 0.091 | 0.828 |
| Li22-35 | PopA | CA **15** 16 18 19 20 21 | 15 18 19 20 21 | 41 (51) | 4 (6) | 0.491 (0.562) | 0.122 (0.118) | 0.754 (0.792) |
|  | PopB | CA **9** | 9 | 11 | 1 | 0.000 | 0.000 | 0.000 |
| Li23-41 | PopA | GT 16 **17** 18 | 16 17 18 | 41 (51) | 2 (3) | 0.137 (0.182) | 0.146 (0.118) | -0.067 (0.355) |
|  | PopB | GT 12 **14** | 12 14 | 11 | 2 | 0.416 | 0.000 | 1.000 |
| Lm2TG | PopA | TG 15 21 22 23 24 25 **26** 27 | 15 21 22 23 24 25 26 27 | 41 (51) | 8 (8) | 0.754 (0.754) | 0.244 (0.196) | 0.679 (0.742) |
|  | PopB | TG 9 **10** | 9 10 | 11 | 2 | 0.247 | 0.091 | 0.643 |
| Lm4TA | PopA | TA 9 10 **11** 12 13 14 15 | 10 11 12 13 14 | 41 (51) | 6 (7) | 0.692 (0.717) | 0.195 (0.196) | 0.721 (0.728) |
|  | PopB | TA **9** 15 20 | 20 | 11 | 3 | 0.658 | 0.000 | 1.000 |
| Li71-5/2 | PopA | CA 8 **9** |  | 41 (51) | 1 (2) | 0.000 (0.039) | 0.000 (0.000) | 0.000 (1.000) |
|  | PopB | CA 8 **9** |  | 11 | 2 | 0.173 | 0.000 | 1.000 |
| LIST7039 | PopA | CA 13 **14** 16 | 13 16 | 41 (51) | 3 (3) | 0.404 (0.388) | 0.049 (0.039) | 0.881 (0.900) |
|  | PopB | CA **17** | 17 | 11 | 1 | 0.000 | 0.000 | 0.000 |
| Li71-33 | PopA | TG **11** 12 | 12 | 41 (51) | 1 (2) | 0.000 (0.020) | 0.000 (0.020) | 0.000 (0.000) |
|  | PopB | TG **11** |  | 11 | 1 | 0.000 | 0.000 | 0.000 |
| Li71-7 | PopA | CA 7 9 12 **13** | 9 12 13 | 41 (51) | 4 (4) | 0.241 (0.244) | 0.073 (0.078) | 0.699 (0.681) |
|  | PopB | CA 7 8 **11** | 8 11 | 11 | 3 | 0.394 | 0.273 | 0.318 |
| CS20 | PopA | TG 17 **20** 21 | 17 20 21 | 41 (51) | 3 (3) | 0.445 (0.426) | 0.024 (0.020) | 0.946 (0.954) |
|  | PopB | TG 12 **15** | 12 15 | 11 | 2 | 0.091 | 0.091 | 0.000 |
| Li45-24 | PopA | CA 8 10 14 **15** 16 | 10 14 15 16 | 41 (51) | 5 (5) | 0.528 (0.577) | 0.122 (0.098) | 0.771 (0.831) |
|  | PopB | CA **7** 8 | 7 | 11 | 2 | 0.519 | 0.000 | 1.000 |
| TubCA | PopA | CA **9** 13 | 9 | 41 (51) | 1 (2) | 0.000 (0.038) | 0.000 (0.000) | 0.000 (1.000) |
|  | PopB | CA 10 **13** | 10 | 11 | 2 | 0.506 | 0.091 | 0.828 |
| LIST7031 | PopA | CA 8 10 **11** | 10 11 | 41 (51) | 3 | 0.139 (0.113) | 0.049 (0.039) | 0.651 (0.655) |
|  | PopB | CA **8** |  | 11 | 1 | 0.000 | 0.000 | 0.000 |
| Overall | PopA |  |  | 41 (51) | 3 (3.47) | 0.259 (0.273) | 0.068 (0.061) | 0.738 (0.777) |
|  | PopB |  |  | 11 | 1.80 | 0.269 | 0.042 | 0.848 |
