# Supplementary material for: Multilocus microsatellite typing (MLMT) reveals host-related population structure in Leishmania infantum from northeastern Italy
Source: PLoS Negl Trop Dis. 2018 Jul 5;12(7):e0006595. doi: 10.1371/journal.pntd.0006595 (PMC6057669; doi:10.1371/journal.pntd.0006595)
Supplement: S1 Protocol — (DOCX) [file pntd.0006595.s006.docx]

**Sequencing of *Leishmania infantum* MHOM/TN/1980/IPT1**

Fifteen microsatellite loci of the WHO reference strain *L. infantum* MHOM/TN/1980/IPT1 were sequenced using the following protocol.

Thirteen out of 15 loci of this reference strain were amplified with new primer pairs, designed by PerlPrimer v1.1.21 software [1], while the same primers of MLMT were used for the remaining LIST7031 and LIST7039 loci (Table 1). The new primers were designed in order to have a longer fragment easier to be sequenced.

PCRs were performed in 30 μL reaction mixtures using the GoTaq® Hot Start Colorless Master Mix (Promega, Madison, USA). The PCR conditions were 95 °C for 2 min, followed by 35 cycles at 94 °C for 1 min, 54 °C for 30 s and 72 °C for 1 min, then 5 min at 72 °C before cooling at 4 °C. The amplicons were purified with the Agencourt® AMPure® XP PCR Purification Kit (Beckman Coulter Inc., Indianapolis, USA) and sequenced in both directions by CEQ 8000 sequencer using the GenomeLab DTCSQuick Start Kit (Beckman Coulter Inc., Indianapolis, USA). The results were analyzed and assembled using the ‘Sequencing’ and ‘Investigator’ packages of CEQ 8000 v.8.0 software.

Sequences of more than 200 bp were submitted to GenBank at NCBI (AN: MG463110-MG463119).

**Table 1. Forward and reverse primers used for direct sequencing of the 15 microsatellite loci of *L. infantum* MHOM/TN/1980/IPT1.**

| **Locus name** | **Forward primer** | **Reverse primer** |  |
| --- | --- | --- | --- |
| Li41-56 | AAAGACCCGAGATGAAAGCC | GAAAGATAGAAGAGGGTATATGCG | n |
| Li46-67 | AGGAGAACGAGGGAAAGGAG | GAAACATGAAGCGAACAACC | n |
| Li21-34 | CCAAGACGATATCCACGATCAC | CTCTTCTCTACACCGCATCG | n |
| Li22-35 | CTTCTACGCTTGATCTTCGG | TTCTCCTTTGTAGTTGTTGC | n |
| Li23-41 | ATTGCTGAGTGCTGCTAGTG | CTTCTACCCTCTTCTCTGCG | n |
| Lm2TG | TTCTGTTGTACGCCACATCG | CCAGATCACAAGTGTGCCAG | n |
| Lm4TA | GATAGCTCCCTATATTTCACG | GTACGCTTCCTACATCATGG | n |
| Li71-5/2 | AGTGCGACAAAGGAACTTGAC | GCTCAACTCTACCATTGTTTCG | n |
| LIST7039 | CTCGCACTCTTTCGCTCTTT | GAGACGAGAGGAACGGAAAA |  |
| Li71-33 | TACTGAGTCGCATCTTTCCC | GGTCAAAGAGTGGATTCCTG | n |
| Li71-7 | TTACCTTGTCCTTGTTCCTG | GTCTGTCTCCATAGCGTGAG | n |
| CS20 | TTCCATAGATCAACCTCTCATCTC | GTTTCGTTGTGTACCGTTGC | n |
| Li45-24 | AGAAAGAACGAAGGAAACCA | CGGGTAGGGAGAAGAATGAC | n |
| TubCA | ACATACGCACAGAGTTCACG | TAGAAGCCGAAGAATGCAGG | n |
| LIST7031 | CCACTGGTGGAAATAGAAAGACT | GGAGAACTAAAACGAGCAGCA |  |

n, New primer pairs designed according to the sequence of *L. infantum* JCPM5 reference strain (A.N.: FR796433.1 to FR796468.1)

**References**

1. Marshall OJ. PerlPrimer: cross-platform, graphical primer design for standard, bisulphite and real-time PCR. Bioinformatics 2004; 20(15): 2471–2472. PMID: 15073005.
